# Evaluating Virtual Reality Patient Education in Cardiac Surgery: Impact on Preoperative Anxiety and Postoperative Patient Satisfaction

**DOI:** 10.3390/jcm13216567

**Published:** 2024-10-31

**Authors:** Sulayman el Mathari, Lieke Kuitert, Noor Boulidam, Saadullah Shehadeh, Robert J. M. Klautz, Robert de Lind van Wijngaarden, Jolanda Kluin

**Affiliations:** 1 Department of Cardiothoracic Surgery, Amsterdam University Medical Center, 1105 AZ Amsterdam, The Netherlands; l.kuitert@amsterdamumc.nl (L.K.); n.boulidam@amsterdamumc.nl (N.B.); s.shehadeh@amsterdamumc.nl (S.S.); r.klautz@amsterdamumc.nl (R.J.M.K.); r.a.f.delindvanwijngaarden@amsterdamumc.nl (R.d.L.v.W.); 2 Department of Cardiothoracic Surgery, Erasmus University Medical Center, 3015 GD Rotterdam, The Netherlands; j.kluin@erasmusmc.nl

**Keywords:** virtual reality patient education, cardiac surgery, preoperative anxiety, patient satisfaction, quality of care

## Abstract

**Background/Objectives**: Preoperative anxiety in cardiac surgery patients can adversely affect mental well-being and postoperative outcomes. Virtual reality (VR) patient education has been proposed as a novel method to enhance patient education and potentially reduce preoperative anxiety. The VR Patient Journey Trial aimed to evaluate the impact of VR patient education on preoperative anxiety and patient satisfaction compared to traditional education methods. **Methods**: This randomized controlled trial included 121 patients undergoing cardiac surgery. Participants were randomized to receive either VR patient education (intervention group) or traditional education (control group). Preoperative anxiety was measured using the State–Trait Anxiety Inventory (STAI) and the Amsterdam Preoperative Anxiety and Information scale (APAIS). Patient satisfaction was assessed postoperatively through a custom questionnaire. Statistical analyses included linear regression and non-parametric testing. **Results**: Neither STAI nor APAIS scores showed differences in preoperative anxiety between both groups. However, the intervention group reported significantly higher patient satisfaction with the information provided (median score 9 vs. 8; *p* < 0.001). Furthermore, women reported higher levels of anxiety than men (*p* = 0.01), and open-ended feedback from participants indicated a need for more detailed information on postoperative rehabilitation and potential risks. **Conclusions**: The VR Patient Journey Trial revealed that, although VR patient education did not significantly reduce preoperative anxiety levels, it significantly improved patient satisfaction with the information provided. These results suggest that VR patient education can be a valuable addition to preoperative patient programs.

## 1. Introduction

Patients undergoing cardiac surgery often experience stress about their awaiting surgical procedure, which can manifest as preoperative anxiety [1]. This anxiety affects the patients’ mental well-being and can also lead to somatic complications after surgery [2]. Anxiety has been linked to heightened activation of the autonomic nervous system and hypothalamic–pituitary–adrenal (HPA) axis, leading to elevated stress hormones levels [3]. Consequently, individuals experiencing anxiety may exhibit an increased heart rate (HR) and blood pressure [4]. Furthermore, patients with persistent anxiety may be susceptible to a chronic state of inflammation due to the augmented release of cytokines, further advancing the development of atherosclerosis [5]. Additionally, there is an observed increase in platelet reactivity, posing a greater risk for the formation of blood clots [6]. All these pathophysiological mechanisms can have significant postoperative implications. Complications include prolonged recovery periods, increased pain experience, hemodynamic instability, elevated mortality rates and lower postoperative patient-satisfaction, highlighting the importance of addressing preoperative anxiety [7,8].

To address preoperative anxiety, various strategies have been employed over the past decades, including both pharmacological and non-pharmacological interventions [9,10,11,12]. Comprehensive patient education, in particular, has been linked to promising results [9,13]. Recent research has demonstrated the effectiveness of patient education using virtual reality (VR) for preoperative anxiety reduction [14,15,16,17]. VR is used here as an immersive and interactive educational tool and has already been introduced in several medical fields globally with the aim of enhancing patient education.

Several studies in cardiology, however, have investigated the impact of VR patient education on preprocedural anxiety and demonstrated inconsistent results. Among these, a study focused on patients undergoing percutaneous atrial septal closure found that VR patient education could potentially alleviate preprocedural anxiety [18]. However, in contrast, another recent study observed no differences in preprocedural anxiety levels between patients exposed to VR patient education and those receiving traditional patient education, in the context of patients awaiting catheter ablation for atrial fibrillation [19].

Despite the incongruence of existing literature on this topic, VR patient education remains largely unexplored in the field of cardiac surgery. To address this gap, we conducted the VR Patient Journey Trial with the aim of investigating the effect of VR patient education in reducing preoperative anxiety and improving postoperative patients’ satisfaction among individuals undergoing cardiac surgery compared to traditional patient education. Preoperative anxiety is measured by the validated Amsterdam Preoperative Anxiety and Information scale (APAIS) [20] and the State-Trait Anxiety Inventory (STAI) [21] Questionnaires. Postoperative patient satisfaction is measured by a custom-made questionnaire. Our hypothesis posits that VR patient education reduces preoperative anxiety and enhances patient satisfaction by preoperatively familiarizing patients with their clinical trajectory before its actualization.

## 2. Methods

The VR Patient Journey Trial is a prospective single-center randomized controlled trial (RCT) hosted by the department of Cardiothoracic Surgery at the Amsterdam University Medical Center (AmsterdamUMC, Amsterdam, The Netherlands). The study population consisted of 100 participants that underwent cardiac surgery in the AmsterdamUMC between April 2023 and July 2024.

### 2.1. Ethics Approval

This study adhered to the principles outlined in the Declaration of Helsinki (2013 version, Fortaleza, Brazil) and complied with the regulations of the Medical Research Involving Human Subjects Act (WMO). Approval for this study was obtained from the Medical Ethics Committee (METC) of the AmsterdamUMC under the reference number W22_214 #22.265. This study was registered on ClinicalTrials.gov with identification number NCT06001489.

### 2.2. Inclusion and Exclusion Criteria

For participation, patients were required to meet specific inclusion criteria, including undergoing cardiac surgery involving coronary artery bypass grafting (CABG) and/or a heart valve procedure performed via sternotomy. Furthermore, patients were required to be at least 18 years old and provide signed informed consent. The exclusion criteria comprised a history of previous cardiac surgery, (concomitant) aortic surgery, cardiac surgery for congenital heart defects, hearing or visual impairments, language barriers (inability to understand, speak or read Dutch), claustrophobia, and facial wounds. An overview of all inclusion and exclusion criteria can be found in Table 1.

### 2.3. Participant Recruitment

Participants were recruited from the surgical waiting list. Patients scheduled for an informed consent visit at the outpatient clinic with their cardiothoracic surgeon were contacted by phone to inquire about their interest in participating. Upon verbal confirmation of interest, participants signed an informed-consent form at the outpatient clinic during their visit. Participants had the right to withdraw from the study at any time and for any reason without facing any consequences.

### 2.4. Randomization and Blinding

Patients were allocated randomly to either the intervention or control group using block randomization with blocks of 4 and a randomization ratio of 1:1. This allocation took place after the patients gave verbal confirmation of interest to participate in this study. Due to the nature of comparing the effects of a physical device to conventional care, blinding of either patients or researchers was not feasible.

### 2.5. Intervention

**Figure 1:**
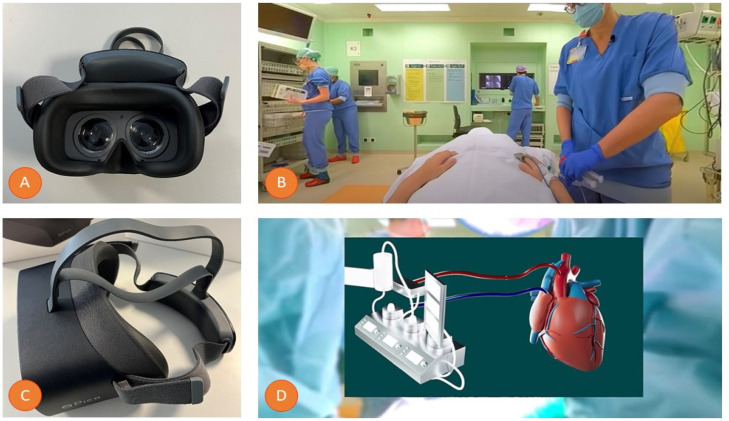
Overview of virtual reality (VR) hardware and intervention. (**A**) This image shows the Pico G2 4K VR headset. (**B**) Screenshot from the 360° VR Patient Journey video in the operating room (OR) that was used during the VR intervention. (**C**) This image shows the Pico G2 4K VR headset from the side. (**D**) Screenshot from the informative three-dimensional (3D) animation incorporated in the 360° VR Patient Journey video. VR = virtual reality; OR = operating room 3D = three-dimensional.

The intervention entailed an immersive educational 360° VR tour, guiding patients through every stage of their clinical journey, spanning from hospital admission, through the surgical procedure, and culminating in clinical discharge. This experience was facilitated using a Pico G2 4K VR headset (Figure 1). The VR tour itself was created by a multidisciplinary team comprising cardiothoracic surgeons, nurses, researchers, and a dedicated video production crew, who recorded and replicated all facets of the anticipated clinical pathway, ensuring a comprehensive simulation of the patient’s forthcoming experience. Moreover, the VR patient-education tour included a three-dimensional (3D) animation that provided a detailed explanation of the surgical procedure. A representative video of this VR tour can be found in Supplemental S1. The control group received traditional patient education by oral information from the attending cardiothoracic surgeon and an informative flyer outlining the procedure.

**Figure 2:**
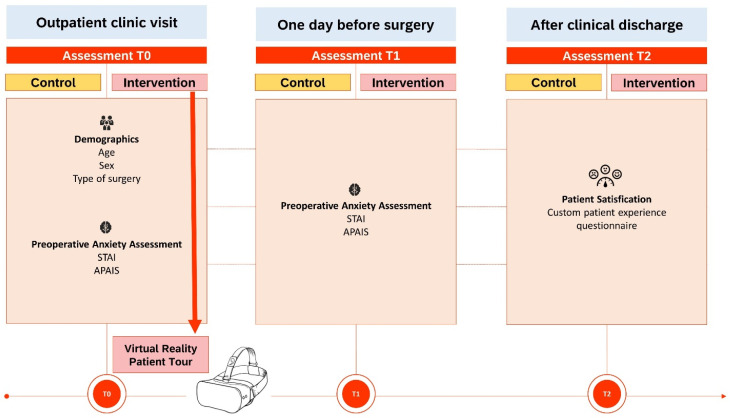
Flowchart showing the study-outline of the VR Patient Journey Trial. APAIS = Amsterdam Preoperative Anxiety and Information scale; STAI = State–Trait Anxiety Inventory; T0 = Baseline; T1 = follow-up 1; T2 = follow-up 2.

After traditional patient education, patients provided written informed consent and were informed whether they were allocated to the intervention or control group. The intervention group received additional VR patient education. Following their outpatient clinic visit, both groups completed two validated questionnaires involving the APAIS [20] and the STAI [21]. These data were considered the ‘baseline’. One day prior to the surgery, during hospital admission, patients were asked to complete the same questionnaires again. These data were referred to as ‘follow-up (FU) moment’. Following their surgical procedure and subsequent discharge from the hospital, patients were provided with a custom patient-satisfaction questionnaire. This questionnaire is designed to evaluate the patients’ overall satisfaction regarding the patient education they received and their experience throughout their actual clinical pathway. The exact study outline is portrayed in Figure 2.

### 2.6. Study Outcomes

The main outcomes of this study are (1) preoperative anxiety at the outpatient clinic measured by the APAIS and STAI questionnaire, (2) preoperative anxiety at the day before surgery assessed by the APAIS and STAI questionnaire, and (3) patient satisfaction after clinical discharge by a custom questionnaire about the quality of patient education and the actual experienced clinical path.

#### 2.6.1. Amsterdam Preoperative Anxiety and Information Scale

The first questionnaire employed was the APAIS (Supplemental S2), consisting of six items and also utilizing a Likert-scale scoring system [20]. In addition to evaluating pre-procedural anxiety (four items; range of 4–20), this questionnaire also assesses the patients’ need for information (two items; range of 2–10), which is associated with their preferred coping style. A score of 11 on the first four items indicates the classification of patients as ‘anxious’.

#### 2.6.2. State–Trait Anxiety Inventory

Spielberger’s STAI questionnaire (Supplemental S3) comprises two distinct scales that provide insight into two types of anxiety: state anxiety and trait anxiety [21]. While trait anxiety refers to the general tendency of an individual to respond to threats in the environment as a stable aspect of personality, state anxiety primarily refers to a temporary emotional state of an individual at a particular moment in time. The latter is situational and can fluctuate based on the environment or specific circumstances. The STAI evaluates both these components, consisting of 40 self-reported items presented on a Likert scale, resulting in a weighted scoring system with a range of 20–80. Higher scores indicate elevated levels of anxiety. In this study, a cut-off value of ≥40 classifies patients as ‘anxious’.

#### 2.6.3. Patient Satisfaction Questionnaire

A customized non-validated questionnaire comprising 10 items was developed to evaluate patients’ satisfaction about the patient education they received and their experience throughout the clinical pathway (Supplemental S4). This questionnaire assessed how well prepared patients felt, whether they had a good understanding of the awaiting clinical pathway and the technical surgical procedure, and whether they still had a feeling of missing any information before their clinical admission. The questionnaire had a score range of 9–50 to quantify responses. Additionally, the questionnaire included an initial open-ended question aimed at collecting qualitative feedback from patients regarding potential areas for enhancement in their educational experience.

#### 2.6.4. Statistical Methods

Descriptive data were presented as numbers with percentages, means with corresponding standard deviations, or medians with interquartile ranges (IQRs) as appropriate. Normality of the baseline characteristics was assessed by means of a Shapiro–Wilk test. Comparisons between groups at baseline were conducted using independent Student *t*-tests for normally distributed data or Mann–Whitney U tests for non-normally distributed data. Categorical data were analyzed using Chi-square testing. Linear regression analyses were employed to explore differences in anxiety at FU between groups, adjusting for baseline anxiety and potential confounding factors. To evaluate the assumption of normality for the residuals for the linear regression model, Quantile–Quantile (Q–Q) plots and histograms were generated. Differences between sex were assessed by means of non-parametric Man-Whitney U testing. For all analyses, a significance level of *p* < 0.05 was applied. All statistical analyses were performed using SPSS 28.0 (SPSS, Inc., Chicago, IL, USA).

## 3. Results

### 3.1. Baseline Characteristics

In total, 121 patients scheduled for cardiac surgery through sternotomy were enrolled in this RCT. The mean ages of patients in the intervention and control group were 67.88 ± 8.56 and 67.08 ± 8.30 years, respectively. This study consisted of 98 male patients, accounting for 81% of participants. Median duration between baseline visit and FU was 24 days (IQR 21) in the VR group and 23.5 days (IQR 21) in the control group. Baseline characteristics did not exhibit differences between the two groups (Table 2). The majority of participants was scheduled for either coronary artery bypass grafting (CABG, N = 48) or aortic valve replacement (AVR, N = 26), with some cases involving concomitant cardiac surgical interventions.

The FU rate for the STAI and APAIS for the study was 81.6% in the VR group, as opposed to 83.6% in the control group. This was mainly due to emergency surgeries, last-minute-planned admissions, and patients unwillingly to further participate. Postoperatively, 117 out of 121 of participants filled out the patient satisfaction survey; 3 patients died post-operatively, and 1 patient was lost at FU.

### 3.2. State–Trait Anxiety Inventory

At baseline, there were no differences in STAI State anxiety between both groups (control group: 39.21 ± 8.76 vs. intervention group: 38.00 ± 11.99; *p* = 0.23). Neither was there a difference in STAI Trait anxiety scores between the groups (control group: 30.3 ± 6.97 vs. intervention group: 29 ± 10; *p* = 0.58) (Table 3 and Table 4). This suggests that the majority of patients did not experience anxiety during their first preoperative outpatient clinic visit, as only patients with scores above 40 were classified as anxious.

At the FU moment during preoperative clinical admission, both State and Trait anxiety scores slightly increased in both the control group and intervention group (Table 3). There were no significant differences within groups compared to the baseline measurement. Also, between groups assessment at FU, performed by non-parametric testing, revealed no significant differences in both State and Trait anxiety (Table 4).

Additional linear regression analyses were performed to examine factors contributing to anxiety levels at FU (Table 5). A single, unadjusted analysis reported no significant differences in anxiety scores between the control and intervention group. Further adjustment for confounding variables, including baseline state anxiety, sex, age and number of days between the first and second measurement did not reveal any significant differences either.

When comparing anxiety scores between men and women, irrespective of group allocation, significant differences were observed at both baseline and FU. At baseline, women reported an average of 8.4 points higher State anxiety compared to men (*p* = 0.01). At FU, women still reported significantly higher state anxiety, averaging 5.3 points more than men (*p* = 0.04) (Table 6). No significant effect modification by sex was observed.

### 3.3. Amsterdam Preoperative Anxiety and Information Scale

APAIS anxiety scores were comparable for both groups, both at baseline (control group: 6 [IQR 6] vs. intervention group: 6 [IQR 4]; *p* = 0.29) and at FU (control group: 5 [IQR 4] vs. intervention group: 5.5 [IQR 5]; *p* = 0.77) (Table 4). Within group differences between the baseline and FU measurements are demonstrated in Table 7.

Also, for the APAIS need-for-information scale, both groups exhibited comparable scores at baseline (mean score control group 5.51 ± 2.45 versus intervention group 5.15 ± 2.52, *p* = 0.47) and FU (mean score control group 4.90 ± 2.13 versus intervention group 4.96 ± 2.61, *p* = 0.83).

When adjusting for confounding factors, there were no significant differences (Table 5). When comparing APAIS anxiety and need-for-information scores between men and women, irrespective of group allocation, significant differences were found for anxiety scores but not for need-for-information (Table 6). Women reported 3.6 APAIS anxiety points higher at baseline (*p* = 0.01) and 3.5 points higher at FU (*p* < 0.01). In contrast to other outcomes of interest, no significant differences between men and women were observed at baseline (*p* = 0.67) or follow-up (*p* = 0.55) with respect to their need for information.

### 3.4. Patient Satisfaction

**Figure 3:**
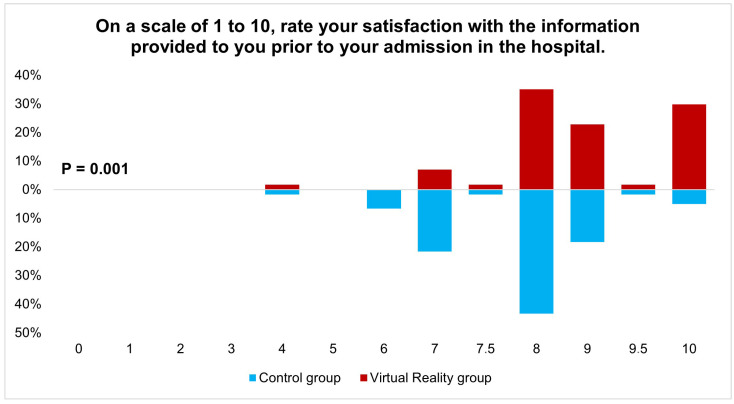
Post-operative patient-satisfaction rates for the control and virtual reality group.

Overall, patients expressed high satisfaction with the quality of information provided during the outpatient clinic visit, with a median satisfaction score of 8 out of 10 (IQR 1). When stratified by group allocation, the intervention group demonstrated significantly higher satisfaction levels compared to the control group, with median scores of 9 (IQR 2) and 8 (IQR 1.8), respectively (*p* < 0.001), as illustrated in Figure 3. These findings suggest that the VR tour constitutes a valuable enhancement to traditional preoperative patient information.

**Figure 4:**
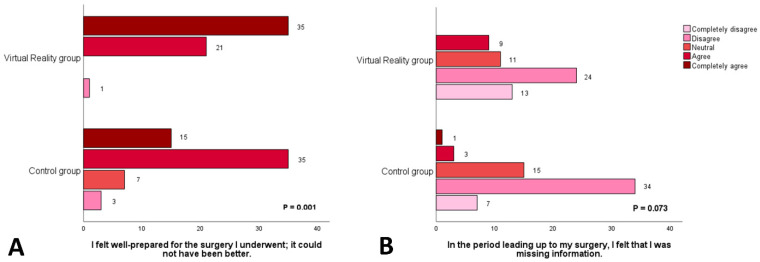
Results from two separate questions of the patient satisfaction questionnaire for both the VR and control group on how well-prepared patients felt to be prior to surgery (**A**) and whether they felt like they were still missing any information before their clinical admission (**B**).

**Figure 5:**
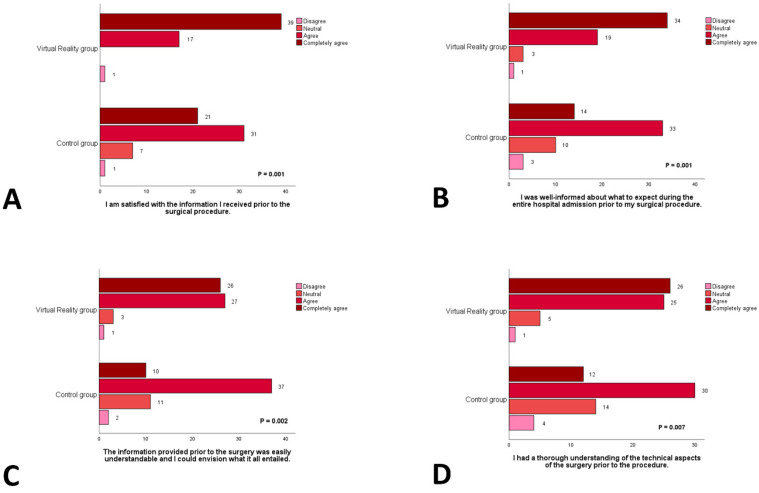
Results from four questions of the patient satisfaction questionnaire for both the VR and control group on satisfaction about the information prior to surgery (**A**), feeling well informed about what to expect during the entire hospital admission (**B**), the comprehensibility of the provided information (**C**), and the understanding of the technical aspects of the awaiting surgical procedure (**D**).

This conclusion is further substantiated by additional components of the patient satisfaction questionnaire (Figure 4 and Figure 5). Within the intervention group, 98% of participants (n = 56) affirmed that they felt adequately prepared for surgery and that the information provision was optimal. This proportion is significantly higher than that of the control group, where only 83% (n = 50) of participants reported similar sentiments (*p* = 0.001). Furthermore, participants in the intervention group reported greater overall satisfaction with the information provided (*p* = 0.001), better expectations regarding the details of hospital admission (*p* = 0.001), enhanced clarity and comprehensibility of the information (*p* = 0.002), and a superior understanding of the technical aspects of the surgical procedure (*p* = 0.007).

Another section of the questionnaire featured an open-ended query regarding potential enhancements to the current patient education program. A total of twenty-three participants provided improvement suggestions: eight from the VR group and fifteen from the control group. Across both groups, participants highlighted a lack of information on the postoperative rehabilitation process (n = 3), the technical details of the surgical procedure (n = 3), and essential intraoperative procedures such as the insertion of a bladder catheter and multiple arterial or venous lines (n = 1). Additionally, 2 participants (1 from each group) indicated they felt insufficiently prepared to manage their surgical wounds postoperatively.

In the control group, five participants recommended that more comprehensive pre-operative information on potential risks and complications following cardiac surgery would be valuable. Specific concerns included surgical wound infections, pneumothorax, arrhythmias, and urinary tract infections related to bladder catheterization. In the intervention group, one participant noted the absence of information regarding the implications of anticoagulation therapy in the context of mechanical heart valve selection. Another participant mentioned a lack of awareness about the importance of physical fitness prior to surgery.

Among the control group, three participants expressed a need for enhanced psychological support following cardiac surgery. One control group participant also suggested that incorporating the VR tour into the outpatient clinic visit could be advantageous. Further recommendations from the control group included providing more information on alternative interventions to cardiac surgery (n = 1), offering an additional outpatient clinic visit (n = 1), and improving pain management education (n = 1).

## 4. Discussion

The VR Patient Journey Trial evaluated the effectiveness of VR patient education in reducing preoperative anxiety and enhancing patient satisfaction among individuals undergoing cardiac surgery. The main findings were that 1) VR patient education significantly increased patient satisfaction, but 2) it had no measurable effect on reducing preoperative anxiety.

### 4.1. Preoperative Anxiety

Contrary to our hypothesis, the results revealed no significant differences in preoperative anxiety levels between the intervention group and the control group, as measured by both the STAI and the APAIS. This outcome aligns with previous RCTs that reported no effect of VR patient education on preprocedural anxiety in cardiac patients with a comparable sample size [19,22]. The lack of significant anxiety reduction in our study might be attributed to the fact that anxiety levels in our population were generally low at baseline. To classify patients as anxious, cut-off values of 40 for the STAI and 11 for the APAIS were employed. Upon examining the data, it was observed that the mean anxiety scores were below these threshold values, indicating that the study population might not have experienced substantial anxiety initially. This contrasts with previous studies, which have suggested that the overall prevalence of pre-procedural anxiety ranges from 64% to 84% [1,23]. This discrepancy may be attributed to cultural differences in anxiety perception, as the earlier studies were conducted in Pakistan and Spain, while our study focused on a Dutch population.

Nevertheless, other earlier RCTs did report a significant effect of VR patient education on reducing preprocedural anxiety [18,24,25]. However, it is important to mention that these RCT’s had a relatively smaller sample size (n = 60, n = 64 and n = 33), possibly overestimating this effect, and were performed in interventional cardiology rather than cardiac surgery, and most of these studies did not use validated questionnaires to assess anxiety.

In line of existing literature [26], the challenge of accurately objectifying anxiety through validated self-reported questionnaires was underscored during the conduct of this study. Despite some patients verbally expressing concerns related to their social situations—such as being a single parent or caregiver for dependent ill family members—these individuals still reported low anxiety levels on the validated questionnaires. This discrepancy suggests that patients may have downplayed their anxiety, potentially providing socially desirable responses rather than accurately reflecting their true emotional state regarding the impending procedure.

### 4.2. Sex Differences in Anxiety

A notable side-finding of this study was the significant difference in anxiety levels between men and women, irrespective of group allocation. Women consistently reported higher levels of state anxiety at both baseline and follow-up, which is consistent with existing literature on sex differences in anxiety [27,28]. This difference could be attributed to women’s greater willingness to express their emotions, whereas men may be more inclined to downplay their feelings of anxiety. Alternatively, it is possible that women experience a stronger anxiety response compared to men [29]. In the latter case, this finding underscores the need for tailored interventions that address the specific psychological needs of female patients undergoing cardiac surgery that may reduce their preoperative anxiety.

### 4.3. Patient Satisfaction

In contrast to the anxiety outcomes in this study, patient satisfaction was significantly higher in the intervention group compared to the control group. Participants that received VR patient education reported greater overall satisfaction, better expectations regarding hospital admission, improved clarity and comprehensibility of the information, and a superior understanding of the technical aspects of the surgical procedure. To our knowledge, this is the first study to evaluate these parameters in cardiac surgery patients. However, similar findings have been reported in other medical specialties, including plastic surgery, colorectal surgery, and gynecology, where VR patient education also led to increased patient satisfaction [15,16,17]. This suggest that VR patient education can enhance the overall preoperative experience by providing a more engaging and comprehensive educational modality.

Additionally, the open-ended feedback from participants in this study identified several important areas for enhancing their patient education in general. Participants expressed a need for more detailed information on the postoperative rehabilitation process, as well as more comprehensive coverage of potential risks and complications. They also emphasized the importance of postoperative psychological support. These insights underscore critical areas where patient education could be improved to better meet the needs of individuals undergoing cardiac surgery, enhancing their overall experience both before and after surgery.

### 4.4. Limitations

This study has several limitations that should be considered. Firstly, blinding and allocation concealment were not feasible, as the researchers involved in delivering the interventions and conducting the analyses were aware of the group assignments. Additionally, the study’s exclusion criteria limited participation to those proficient in Dutch, thereby excluding patients with foreign ethnic backgrounds. This exclusion might have inadvertently left out a population that could have experienced heightened pre-procedural anxiety due to language barriers and uncertainties about the surgical procedure, potentially affecting the generalizability of the findings. Furthermore, it is important to reflect on the objectivity of the selected outcome measures. Although both the STAI and APAIS questionnaires are widely used, it is essential to acknowledge the potential introduction of self-report bias when using these tools. This concern is particularly relevant when observing the notably low levels of anxiety reported within this cohort. Lastly, it is important to note that our patient satisfaction questionnaire, though tailored to the specific context of this study, remains non-validated. Therefore, the findings derived from this tool should be interpreted with appropriate caution.

### 4.5. Future Directions

Future research should incorporate more objective measures for preoperative anxiety. For example, the utilization of galvanic skin responses and (salivary) cortisol levels could be useful alternative instruments to measure anxiety [30,31]. Moreover, it has been suggested that VR might be of particular interest in specific patients who are generally more familiar with the application of modern multimedia technologies [32,33]. This would suggest that its effectiveness would apply to a specific patient profile rather than the general population. Future studies could help identify particular patient profiles that might derive greater benefit from VR patient education. And future studies should also incorporate multiple languages to encompass a broader range of patients and enhance the generalizability of the findings.

Besides VR, it is fair to acknowledge that various other tools can also reduce preoperative anxiety. These alternative tools include interventions such as conventional video-based education, music therapy or meditation. Like our findings, many of these studies report limited effects on preoperative anxiety, but they consistently demonstrate significant improvements in patient satisfaction [34,35]. This underscores the potential of diverse approaches to deliver more comprehensible information and create a less stressful care experience for patients, ultimately enhancing their confidence and overall satisfaction with care. The selection of the most appropriate tool should be tailored to the specific needs and preferences of the patient, ensuring personalized and effective patient care.

## 5. Conclusions

The VR Patient Journey Trial revealed that, although VR patient education did not significantly reduce preoperative anxiety levels, it significantly improved patient satisfaction with the information provided. These results suggest that VR patient education can be a valuable addition to preoperative patient programs. Future research should focus on employing more objective measures of preoperative anxiety to better understand the potential broader impact of VR patient education on patient preparedness for cardiac surgery.

## Figures and Tables

**Table 1:** Inclusion and exclusion criteria. CABG; coronary artery bypass grafting, ICU; intensive care unit.

Inclusion Criteria	Exclusion Criteria
18 Years of age	History of cardiac surgery
Undergoing cardiac surgery involving CABG and/or heart valve procedure performed via sternotomy	(Concomitant) aortic surgery Congenital heart defects Hearing or visual impairments
Signed Informed Consent form	Inability to speak or read Dutch
	Claustrophobia
	Facial wounds

**Table 2:** The baseline characteristics of the VR Patient Journey Trial.

	VR Group (n = 60)	Control Group (n = 61)	*p*-Value *
Age (years)	67.88 ± 8.56	67.08 ± 8.30	0.60
Sex, male (%)	48 (80)	50 (82)	0.82
Cardiac surgery type			0.36
AVR	12 (20)	19 (31.2)	
AVR + MVR	1 (1.7)	1 (1.6)	
AVR + CABG	9 (15)	3 (4.9)	
AVR + TVR	1 (1.7)	0 (0.0)	
CABG	27 (45)	22 (36)	
CABG + MVR	1 (1.7)	0 (0.0)	
MVR + TVR + CABG	1 (1.7)	0 (0.0)	
MVR + TVR	5 (8.3)	9 (14.8)	
MV Replacement	2 (3.3)	6 (9.8)	
TV Replacement	1 (1.7)	1 (1.6)	
follow-up duration (days)	23.5 (21)	23 (24)	0.77

Data are mean ± standard deviation, median with interquartile range (IQR), n (%). * Group differences were tested with the independent Student *t*-test, Mann–Whitney U-test or Chi-square test. VR = virtual reality, CABG = Coronary Artery Bypass Grafting, AVR = Aortic Valve Replacement, MVR = Mitral Valve Repair, MV Replacement = Mitral Valve Replacement, TVP = Tricuspid Valve Repair, TV Replacement = Tricuspid Valve Replacement, FU = follow-up.

**Table 3:** State–Trait Anxiety Inventory results within groups.

Questionnaires	VR Group				Control Group			
	Baseline (N = 60)	FU (N = 49)	Δ VR Group *	Within-Group *p*-Value **	Baseline (N = 61)	FU (N = 51)	Δ Control Group *	Within-Group *p*-Value **
STAI (State–Trait Anxiety Inventory)								
State Anxiety score (20–80)	38.00 ± 11.99	39.90 ± 11.75	1.82 [−0.88; 4.51]	0.16	39.21 ± 8.76	40.18 ± 9.51	0.78 [−1.20; 2.76]	0.35
Trait Anxiety score (20–80)	29 (10)	27.00 (12)	−1.24 [−2.80; 0.31]	0.13	30.03 ± 6.97	29.65 ± 6.82	−0.10 [−1.63; 1.43]	0.85

Data are mean ± standard deviation or median with interquartile range (IQR). * Δ Intervention group and Δ control group are equal to mean change in score (FU—baseline). ** Within-group differences were investigated using Wilcoxon signed-rank testing. VR = virtual reality, FU = follow-up.

**Table 4:** STAI and APAIS between-groups results.

	Baseline	Follow-Up
	Between Group *p*-Value *	Between Group *p*-Value *
STAI (State–Trait Anxiety Inventory)		
State Anxiety score (20–80)	0.23	0.86
Trait Anxiety score (20–80)	0.58	0.73
APAIS (Amsterdam Pre-Operative Anxiety and Information scale)		
Anxiety score (4–20)	0.29	0.77
Need for information score (2–10)	0.47	0.83

Data are mean ± standard deviation or median with interquartile range (IQR). * Group differences were investigated with Mann–Whitney U testing. FU = follow-up.

**Table 5:** Linear regression analysis STAI and APAIS.

	Single Linear Regression			Multiple Linear Regression *		
	*β* [95% CI]	*p*-Value	R²	*β* [95% CI]	*p*-Value	R²
STAI State Anxiety	−0.28 [−4.51; 3.96]	0.90	<0.001	0.62 [−2.58; 3.82]	0.70	0.47
STAI Trait Anxiety	0.50 [−2.54; 3.53]	0.69	0.001	−0.65 [−2.63; 1.32]	0.51	0.61
APAIS Anxiety	0.28 [−0.89; 1.45]	0.64	0.002	0.29 [−0.73; 1.30]	0.56	0.30
APAIS Need for information	−0.05 [−0.99; 0.87]	0.90	<0.001	−0.11 [−0.95; 0.73]	0.79	0.23

Regression coefficients are presented for the intervention group compared to the control group. * Multiple linear regression analyses are adjusted for baseline scores, age, sex, and number of days before surgery. STAI = State–Trait Anxiety Inventory, APAIS = Amsterdam Pre-Operative Anxiety and Information scale.

**Table 6:** Differences in anxiety scores between men and women.

Questionnaires	Baseline			Follow-Up		
	Male (N = 82)	Female (N = 18)	*p*-Value	Male (N = 82)	Female (N = 18)	*p*-Value
STAI (State–Trait Anxiety Inventory)						
State Anxiety score (20–80)	37.24 ± 9.49	45.61 ± 10.00	0.01	39.09 ± 10.55	44.39 ± 10.07	0.04
Trait Anxiety score (20–80)	29.26 ± 6.34	36.44 ± 13.45	0.09	28.85 ± 6.45	34.61 ± 10.49	0.03
APAIS (Amsterdam Pre-Operative Anxiety and Information scale)						
Anxiety score (4–20)	5.0 (3.0)	8.56 ± 3.01	0.01	5.0 (3.0)	8.50 ± 2.94	<0.01
Need for information score (2–10)	5.27 ± 2.53	5.39 ± 2.40	0.67	4.90 ± 2.47	4.94 ± 2.07	0.55

Data are mean ± standard deviation or median with interquartile range (IQR). Reported *p*-values represent results from Mann–Whitney U-testing.

**Table 7:** Amsterdam Pre-Operative Anxiety and Information scale within groups.

Questionnaires	VR Group				Control Group			
	Baseline (N = 60)	FU (N = 52)	Δ VR Group *	Within-Group *p*-Value **	Baseline (N = 61)	FU (N = 51)	Δ Control Group *	Within-Group *p*-Value **
APAIS (Amsterdam Pre-Operative Anxiety and Information scale)								
Anxiety score (4–20)	6.00 (4)	5.50 (5)	0.12 [−0.88; 1.11]	0.88	6.00 (6)	5.00 (4)	−0.29 [−0.89; 0.30]	0.23
Need for information score (2–10)	5.15 ± 2.52	4.90 ± 2.13	−0.33 [−1.13; 0.48]	0.39	5.51 ± 2.45	4.96 ± 2.61	−0.37 [−1.03; 0.29]	0.21

Data are mean ± standard deviation or median with interquartile range (IQR). * Δ Intervention group and Δ control group are equal to mean change in score (FU—baseline). ** Within-group differences were investigated using Wilcoxon signed-rank testing. VR = virtual reality, FU = follow-up.

## Data Availability

All data from this study is available upon request to the corresponding author.

## Supplementary Materials

The following supporting information can be downloaded at: https://www.mdpi.com/article/10.3390/jcm13216567/s1.

## Abbreviations

Coronary Artery Bypass Grafting	CABG
Virtual Reality	VR
Three Dimensional	3D
Randomized Controlled Trial	RCT
Quality of Recovery 15	QoR-15
Intensive Care Unit	ICU
Medical Research Involving Human Subjects Act	WMO
Medical Ethics Committee	METC
Numeric Rating Scale	NRS
State–Trait Anxiety Inventory	STAI
State–Trait Anxiety Inventory 6	STAI-6
Standard Deviation	SD
Inter-Quartile Range	IQR
