# Supplementary material for: Evaluating Virtual Reality Patient Education in Cardiac Surgery: Impact on Preoperative Anxiety and Postoperative Patient Satisfaction
Source: J Clin Med. 2024 Oct 31;13(21):6567. doi: 10.3390/jcm13216567 (PMC11546597; doi:10.3390/jcm13216567)
Supplement: Supplementary file 1 [file jcm-13-06567-s001.zip › jcm-3212358-supplementary.pdf]

Supplementary files

Supplemental S1. A video of the virtual reality patient tour used in this used at the preoperative outpatient clinic visit as patient education. (377) Bypass operatie van de kransslagaderen - 3D Virtual Reality Patient Tour (AmsterdamUMC) - YouTube

Supplemental S2. The State Trait Anxiety Inventory questionnaire.

SELF-EVALUATION QUESTIONNAIRE STAI Form Y-1

Please provide the following information:

Name____Date_____________S_____

Age___________________ Gender (*Circle*) **M** **F** T_____

DIRECTIONS:

A number of statements which people have used to describe themselves are given below. Read each statement and then circle the appropriate number to the right of the statement to indicate how you feel *right* now, that is, *at this moment*. There are no right or wrong answers. Do not spend too much time on any one statement but give the answer which seems to describe your present feelings best.

	NOT AT ALL	SOMEWHAT	MODERATELY SO	VERY MUCH SO
1. I feel calm	1	2	3	4
2. I feel secure	1	2	3	4
3. I am tense	1	2	3	4
4. I feel strained	1	2	3	4
5. I feel at ease	1	2	3	4
6. I feel upset	1	2	3	4
7. I am presently worrying over possible misfortunes	1	2	3	4
8. I feel satisfied	1	2	3	4
9. I feel frightened	1	2	3	4
10. I feel comfortable	1	2	3	4
11. I feel self-confident	1	2	3	4
12. I feel nervous	1	2	3	4
13. I am jittery	1	2	3	4
14. I feel indecisive	1	2	3	4
15. I am relaxed	1	2	3	4
16. I feel content	1	2	3	4
17. I am worried	1	2	3	4
18. I feel confused	1	2	3	4
19. I feel steady	1	2	3	4
20. I feel pleasant	1	2	3	4

© Copyright 1968,1977 by Charles D. Spielberger. All rights reserved.
Published by Mind Garden, Inc., 1690 Woodside Rd, Suite 202, Redwood City, CA 94061

STAIP-AD Test Form Y
www.mindgarden.com

SELF-EVALUATION QUESTIONNAIRE

STAI Form Y-2

Name____Date__________

DIRECTIONS

A number of statements which people have used to describe themselves are given below. Read each statement and then circle the appropriate number to the right of the statement to indicate how you *generally* feel. There are no right or wrong answers. Do not spend too much time on any one statement but give the answer which seems to describe how you generally feel.

	ALMOST NEVER	SOMETIMES	OFTEN	ALMOST ALWAYS
21. I feel pleasant	1	2	3	4
22. I feel nervous and restless	1	2	3	4
23. I feel satisfied with myself	1	2	3	4
24. I wish I could be as happy as others seem to be	1	2	3	4
25. I feel like a failure	1	2	3	4
26. I feel rested	1	2	3	4
27. I am "calm, cool, and collected"	1	2	3	4
28. I feel that difficulties are piling up so that I cannot overcome them	1	2	3	4
29. I worry too much over something that really doesn't matter	1	2	3	4
30. I am happy	1	2	3	4
31. I have disturbing thoughts	1	2	3	4
32. I lack self-confidence	1	2	3	4
33. I feel secure	1	2	3	4
34. I make decisions easily	1	2	3	4
35. I feel inadequate	1	2	3	4
36. I am content	1	2	3	4
37. Some unimportant thought runs through my mind and bothers me	1	2	3	4
38. I take disappointments so keenly that I can't put them out of my mind	1	2	3	4
39. I am a steady person	1	2	3	4
40. I get in a state of tension or turmoil as I think over my recent concerns and interests	1	2	3	4

© Copyright 1968,1977 by Charles D. Spielberger. All rights reserved.
Published by Mind Garden, Inc., 1690 Woodside Rd, Suite 202, Redwood City, CA 94061

STAIP-AD Test Form Y
www.mindgarden.com

Supplemental S3. The Amsterdam Preoperative Anxiety Inventory Scale.

1. I am worried about the anesthetic.
2. The anesthetic is on my mind continually.
3. I would like to know as much as possible about the anesthetic.
4. I am worried about the procedure.
5. The procedure is on my mind continually.
6. I would like to know as much as possible about the procedure.

The measure of agreement with these statements should be graded on a five-point Likert scale from 1 = not at all to 5 = extremely.

Supplemental S4. Custom patient satisfaction questionnaire used in the VR Patient Journey Trial.

1. I am satisfied with the information I received prior to the surgery.

- Totally agreed
- Agree
- Neither agree nor disagree
- Disagree
- Totally disagreed

2. I felt well-prepared for the surgery I underwent; it couldn't have been better.

- Totally agreed
- Agree
- Neither agree nor disagree
- Disagree
- Totally disagreed

3. I knew well in advance what to expect throughout my entire hospital stay before my surgery.

- Totally agreed
- Agree
- Neither agree nor disagree
- Disagree
- Totally disagreed

4. I knew in advance what would technically happen during the surgery.

- Totally agreed
- Agree
- Neither agree nor disagree
- Disagree

Totally disagreed

5. I still felt that I was missing some information leading up to my surgery.

- Totally agreed
- Agree
- Neither agree nor disagree
- Disagree
- Totally disagreed

6. The information I received leading up to the surgery was easy to understand, and I was able to clearly visualize what it entailed.

- Totally agreed
- Agree
- Neither agree nor disagree
- Disagree
- Totally disagreed

7. The information I received leading up to the surgery was reassuring.

- Totally agreed
- Agree
- Neither agree nor disagree
- Disagree
- Totally disagreed

8. How satisfied are you with the information you received before your clinical admission? Circle the number on a scale from 1 to 10.
1 = Not at all satisfied
10 = Very satisfied
1 2 3 4 5 6 7 8 9 10

9. The virtual reality tour of the entire hospital stay was a valuable addition to the oral information provided by the surgeon. *(only applicable to patients in the intervention group)*

- Totally agreed
- Agree
- Neither agree nor disagree
- Disagree

Totally disagreed

10. Could the information provided before the surgery have been improved? If so, how could it have been enhanced?
(PLEASE PROVIDE OPEN RESPONSE)
